# Naso-Septal Tuberculosis Mimicking Dermoid in a 3-Year old Child

**Published:** 2018-07

**Authors:** Poonam Sagar, Vikram Wadhwa, Ishwar Singh, Varuna Mallya, Pragya Rajpurohit

**Affiliations:** 1 *Department of Otorhinolaryngology, Head and Neck Surgery, Maulana Azad Medical College, Delhi, India.*; 2 *Department of Pathology, Maulana Azad Medical College, Delhi, India.*

**Keywords:** Dermoid cyst, Nose, Tuberculosis

## Abstract

**Introduction:**

Tuberculosis is a communicable disease caused by mycobacterium tuberculosis that primarily affects the lungs. Primary tuberculosis of the nose in the pediatric age group is rare. The diagnosis of this common entity in the present case was challenging.

**Case Report:**

We report the case of a 3-year old girl who presented with a painless swelling over the dorsum of the nose for 7 months. Imaging revealed a mass lesion eroding nasal bones, septum and frontal bone with intracranial extension. Endoscopic examination showed a friable mass in the superior aspect of the nasal septum, extending intracranially. Histopathology confirmed the diagnosis of nasal tuberculosis, and the patient improved on category I anti-tubercular therapy.

**Conclusion:**

Midline nasal swelling in children needs to be differentiated from congenital nasal swelling. A high index of suspicion is required for correct diagnosis of a patient with nasal tuberculosis. Anti-tubercular therapy is the mainstay of treatment.

## Introduction

Tuberculosis in the head and neck region mainly affects the cervical lymph nodes, pharynx, larynx and ear (1). Nasal tuberculosis is rare and is mostly associated with lung tuberculosis. Primary nasal tuberculosis is very rare; indeed Butt reviewed the English-language medical literature over 95 years and found only 35 cases of nasal tuberculosis (2). Nasal tuberculosis presenting as a swelling at the root of the nose in the pediatric age group has not been reported in the English literature. Midline swelling over the nose in the pediatric age group is most commonly attributed to dermoid cysts, gliomas, encephalocoeles, teratomas and hemangiomas. We report the case of nasal tuberculosis in a 3-year old girl who presented with swelling at the root of the nose with an underlying hidden mass in the nasal cavities.

## Case Report

**Fig 1:**
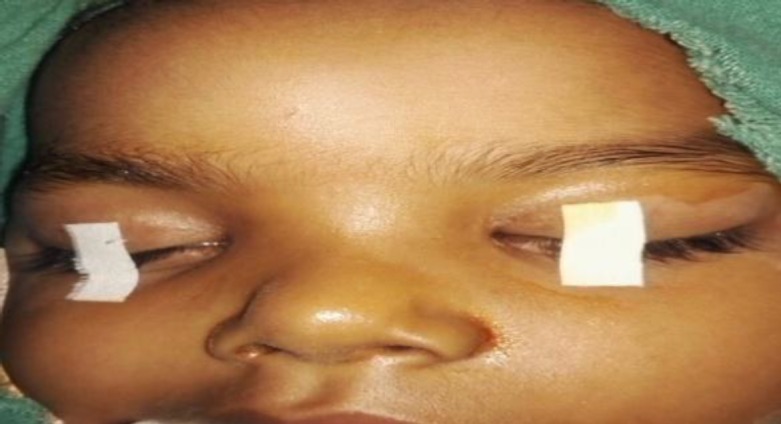
Ill-defined swelling at the root of the nose with broadening of the nasal bridge

A 3-year old girl presented with swelling over the root of the nose. Her parents had noticed swelling 7 months previously, which was gradually progressive and was the size of a small lemon at the time of presentation. The swelling was not associated with fever, nasal obstruction, bleeding or any other nasal complaints. The patient was prescribed antibiotics and anti-inflammatory drugs by the general practitioner, following which the swelling decreased slightly. The patient had recurrent episodes in which the swelling increased in size. The size of the swelling decreased on medication, but never completed resolved. There was no history of tubercular contact or any other family history of tuberculosis. On examination, the root of the nose was broadened by the ill-defined 2.5×2.0 cm swelling, but the overlying skin was normal (Fig.1).

**Fig2:**
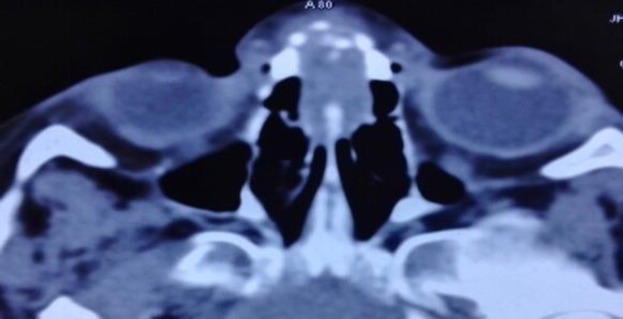
CECT nose (axial section) showing enhancing soft tissue mass lesion in the superior aspect of both the nasal cavities measuring 2.0 × 1.8 × 2.8 cm (AP × TR × CC), eroding intervening nasal septum, frontal and nasal bones (CECT, Contrast enhanced computed tomography).

On palpation, the swelling was soft and non-tender with a normal temperature. Anterior rhinoscopy revealed no abnormality. Blood analysis showed raised erythrocyte sedimentation rate (ESR; 38mm). The patient had a positive Monteux test, and a chest X- ray showed no abnormality. Contrast enhanced computed tomography (CT) of the nose revealed an enhancing soft tissue mass lesion in the superior aspect of both of the nasal cavities, measuring 2.0 ×1.8 × 2.8 cm (AP × TR × CC), eroding the intervening nasal septum, frontal and nasal bones at the glabella and reaching to the subcutaneous tissue (Fig.2). 

**Fig3:**
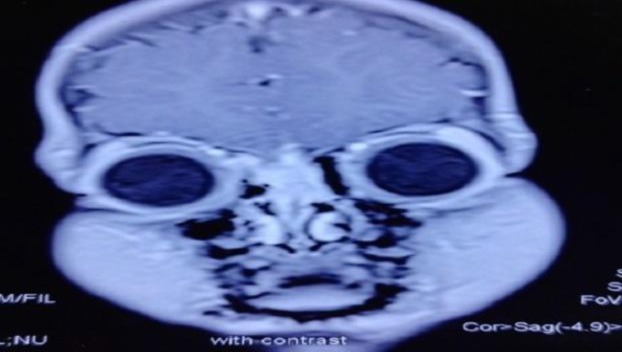
MRI brain and nose (coronal section) showing enhancing soft tissue mass in the basifrontal extra-axial region with dural enhancement extending inferiorly into the superior parts of nasal cavities with osseous destruction (MRI, magnetic resonance imaging).

The base of the skull was eroded with a small intracranial extension. Magnetic resonance imaging (MRI) of the brain and paranasal sinus revealed enhancing soft tissue mass in the basifrontal extra-axial region with dural enhancement extending inferiorly into the superior parts of the nasal cavities with osseous destruction (Fig.3). 

**Fig 4:**
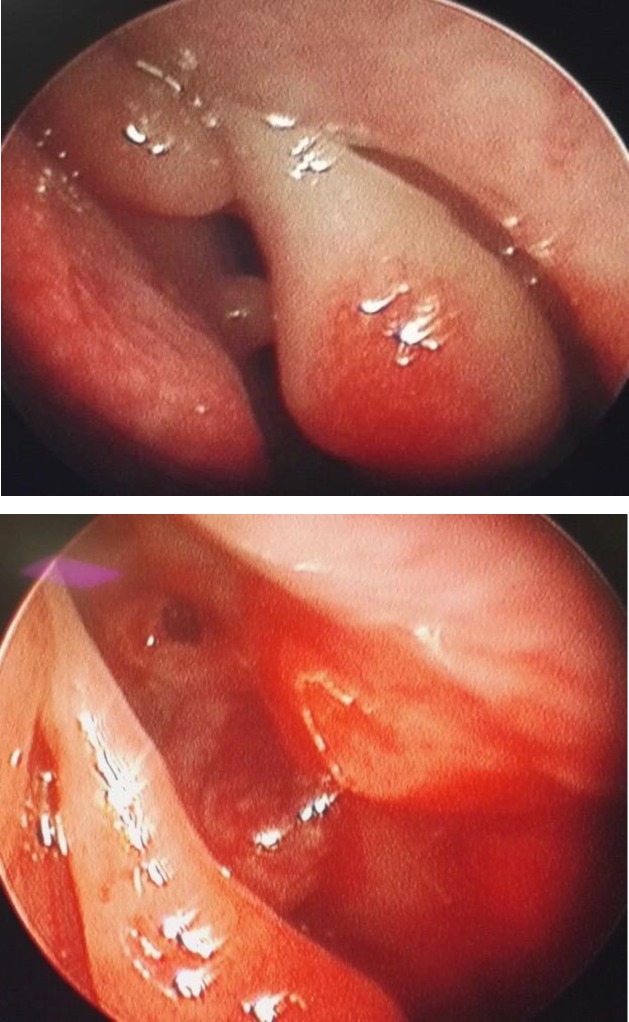
Nasal endoscopic pictures. A- left nasal cavity picture showing smooth, reddish, friable mass in the superior part of the nasal cavity attached to the septum. B- Right nasal cavity showing erosion of superior-most aspect of nasal septum with friable mass extending superiorly towards cranial cavity

The patient underwent endoscopic examination and biopsy under general anesthesia. On endoscopy, a smooth reddish mass was seen in the left nasal cavity. The mass was friable and found to be eroding the nasal septum, reaching the opposite nasal cavity (Fig.4). 

**Fig 5:**
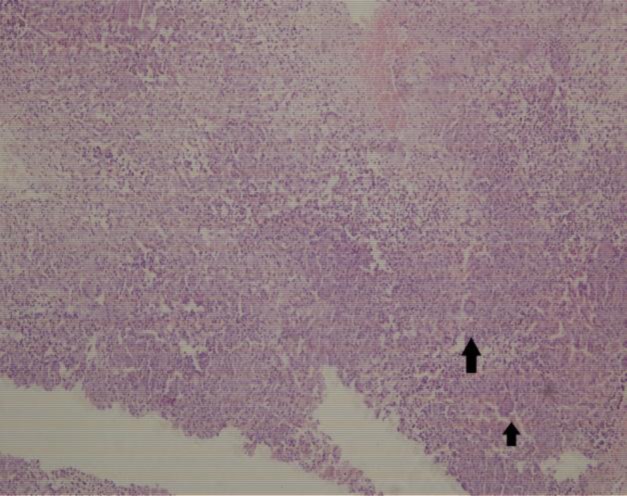
Photomicrograph showing epithelioid giant cell granulomas admixed with Langhans giant cells (black arrows) (H&E ×100)

The mass was also found to be eroding the most superior aspect of the nasal septum with intracranial extension. As the mass was invasive with intracranial extension, a biopsy was taken and further definitive surgery was deferred for final histopathology diagnosis. Histopathology showed lesional tissue focally lined pseudostratified ciliated columnar epithelium with subepithelial epithelioid cell granuloma admixed with Langhans giant cells, suggestive of tuberculosis (Fig.5). 

The patient was started on category I anti- tubercular treatment. Isoniazid, rifampicin, pyrazinamide and ethambutol was prescribed for the first 2 months followed by isoniazid and rifampicin for the next 4 months. The swelling subsided on anti-tubercular therapy. At the time of writing, the patient has completed 6 months of anti-tubercular treatment and has been symptom free for 7 months following anti-tubercular therapy.

## Discussion

Tuberculosis is a communicable disease caused by mycobacterium tuberculosis. The mode of spreading of tuberculosis is a droplet infection, and the lungs are the most common site of infection. Primary nasal tuberculosis is a rare entity (3).

Nasal infection may result from direct inoculation, nose picking, by open tuberculosis or by haematogenous dissemination of a primary infection. Three forms of nasal tuberculosis have been recognized. Lupus vulgaris, or the nodular form, presents as papules and crusting over the nasal vestibule and the adjoining skin. The ulcerative form involves the cartilaginous nasal septum or the inferior turbinate and presents with nasal obstruction, crusting, discharge and epistaxis. The third form is sinus granuloma, which present as a mass in the paranasal sinuses with osteomyelytic changes (1).

In the present case, a 3-year old girl presented with swelling at the root of the nose. Pediatric nasal tuberculosis has previously been reported in the literature at the age of 10–12 years, and the present case is the youngest case of nasal tuberculosis. Nasal tuberculosis has been reported in the literature presenting as nasopharyngeal mass, sino-nasal mass, pansinusitis epistaxis and epilepsy, facial abscess, proliferative lesion over the dorsum of the nose and as nasal bone osteomyelitis with cervical lymphadenitis (4-14). Nasal tuberculosis can occur as primary tuberculosis or as secondary tuberculosis associated with pulmonary tuberculosis (15). The presentation of tuberculosis as a midline nasal swelling in the pediatric age group has not previously been reported to the best of our knowledge.

Midline nasal swelling in the pediatric age group need to be differentiated from nasal dermoids, teratomas, gliomas, encephaloceles and hemangiomas (16). In the present case, swelling was ill-defined with normal overlying skin and did not increase while crying. On contrast enhanced CT of the nose and paranasal sinuses, an enhancing mass was seen in the superior aspect of the nasal cavities, eroding the nasal septum, frontal and nasal bones with erosion of the skull base and small intracranial extension. MRI of the brain and nose showed enhancing soft tissue mass in the basifrontal region with dural enhancement (extra-axial). These features suggest a mitotic etiology. Dermoids are non-enhancing lesions, while hemangiomas and teratomas are enhancing lesions. Gliomas and encephalocoeles are well delineated on imaging.

Nasal endoscopy showed a pale polypoidal, non-pulsatile mass arising from the roof of the nasal cavity and the superior-most part of the nasal septum. The mass did not increase on anesthetic Valsalva maneuver. The nasal mass was found to be friable and eroding the superior-most part of the bony nasal septum, reaching the other nasal cavity with erosion of the cribriform plate with intracranial extension. Unlike the classical description in which nasal tuberculosis involves the cartilaginous part of the nasal septum, our case had involvement of the bony part of the nasal septum. The diagnosis of nasal tuberculosis was confirmed on histopathology, which showed an epithelioid granuloma with Langhans giant cells (Fig.5). In addition to this, the raised ESR and positive Monteux test also supported the diagnosis of tuberculosis. As chest X-ray was within the normal limits, with no signs of tuberculosis of any other site, confirming primary nasal tuberculosis. The present case responded well to category I anti-tubercular treatment according to the revised national tuberculosis control program.

The present case report highlights the uncommon presentation of primary nasal tuberculosis as a midline nasal swelling in the pediatric age group, with the absence of nasal symptoms and the involvement of bone with intracranial extension.
